# Phylogenetic Origin of Primary and Secondary Metabolic Pathway Genes Revealed by *C. maxima* and *C. reticulata* Diagnostic SNPs

**DOI:** 10.3389/fpls.2019.01128

**Published:** 2019-09-24

**Authors:** Milena do Amaral, Marcia Fabiana Barbosa de Paula, Frederique Ollitrault, Ronan Rivallan, Edson Mario de Andrade Silva, Abelmon da Silva Gesteira, François Luro, Dominique Garcia, Patrick Ollitrault, Fabienne Micheli

**Affiliations:** ^1^Centro de Biotecnologia e Genética (CBG), Departamento de Ciências Biológicas (DCB), Universidade Estadual de Santa Cruz (UESC), Ilhéus, Brazil; ^2^CIRAD, UMR AGAP, San Giuliano, France; ^3^CIRAD, UMR AGAP, Montpellier, France; ^4^Embrapa Mandioca e Fruticultura, Departamento de Biologia Molecular, Cruz das Almas, Brasil; ^5^INRA, UMR AGAP, San Giuliano, France

**Keywords:** pummelos, mandarins, KASPar, citrus quality, carotenoids, sugars

## Abstract

Modern cultivated Citrus species and varieties result from interspecific hybridization between four ancestral taxa. Among them, *Citrus maxima* and *Citrus reticulata*, closely associated with the pummelo and mandarin horticultural groups, respectively, were particularly important as the progenitors of sour and sweet oranges (*Citrus aurantium* and *Citrus sinensis*), grapefruits (*Citrus paradisi*), and hybrid types resulting from modern breeding programs (tangors, tangelos, and orangelos). The differentiation between the four ancestral taxa and the phylogenomic structure of modern varieties widely drive the phenotypic diversity’s organization. In particular, strong phenotypic differences exist in the coloration and sweetness and represent important criteria for breeders. In this context, focusing on the genes of the sugar, carotenoid, and chlorophyll biosynthesis pathways, the aim of this work was to develop a set of diagnostic single-nucleotide polymorphism (SNP) markers to distinguish the ancestral haplotypes of *C. maxima* and *C. reticulata* and to provide information at the intraspecific diversity level (within *C. reticulata* or *C. maxima*). *In silico* analysis allowed the identification of 3,347 SNPs from selected genes. Among them, 1,024 were detected as potential differentiation markers between *C. reticulata* and *C. maxima*. A total of 115 SNPs were successfully developed using a competitive PCR technology. Their transferability among all *Citrus* species and the true citrus genera was very good, with only 0.87% of missing data. The ancestral alleles of the SNPs were identified, and we validated the usefulness of the developed markers for tracing the ancestral haplotype in large germplasm collections and sexually recombined progeny issued from the *C. reticulata*/*C. maxima* admixture gene pool. These markers will pave the way for targeted association studies based on ancestral haplotypes.

## Introduction

Citrus is an important food crop worldwide, and its commercialization depends on two main markets: the fruit-processing market and the fresh fruit market. Currently, world citrus production stands at about 52 million tons. Brazil is the main orange producer at 17.3 million tons, followed by China, the European Union and the USA, at 7.2, 6.5, and 5.1 million tons, respectively (Usda, 2019). One-third of total orange production is destined for juice processing. In Brazil, 95% of juice production is destined for the export market, while the USA dedicates its production to the domestic market (86%). In parallel with the fruit juice industry, consumer demand for fresh citrus fruit has increased worldwide. Mandarins and other small citrus are highly consumed and exported from the Mediterranean region, while acid citrus (limes and lemons) and grapefruit reached record production in the last 2 years (2016–2018). In 2018, acid citrus production increased 5%, reaching 8.2 million tons, mainly from Mexico, Argentina, the European Union, and Turkey. Grapefruits also showed significant increases in consumption and exports (3 and 8%, respectively), totaling 7 million tons/year due to crop investment in the USA and China (Usda, 2019).

Regarding the fresh fruit market, characteristics related to fruit quality such as bright fruit color, balanced acidity/sugar ratio, fruit size, peel thickness, peelability, low seed number, and longer shelf life are important for meeting consumer expectations. However, some of these characteristics are highly dependent of the plant genotype and its interactions with the environmental conditions, generating a great challenge for genetic studies and for citrus-breeding programs (Goldenberg et al., 2014).

Another challenge for breeding programs is the phylogenomic complexity of the Citrus genus due to the particularities of its reproductive biology and its wide cultivation history (Ollitrault et al., 2012; Garcia-Lor et al., 2013). The citrus varieties show great sexual intercompatibility, which has led to intergeneric and interspecific hybrids throughout the evolution process of the citrus group (Curk et al., 2016). Many of these hybrids are characterized by spontaneous formation of nucellar embryos, which has contributed to maintaining their genetic stability and perpetuating hybrids as apomictic clones (Ollitrault and Navarro, 2012). For a long time, only the mandarins (*Citrus reticulata* Blanco), pummelos (*Citrus maxima* [Burm.] Merr.), and citrons (*Citrus medica L*.) were considered citrus basal taxa (Scora, 1975; Barrett and Rhodes, 1976). Molecular marker studies have confirmed the important role of these three taxa and showed that the papeda, *Citrus micrantha* Wester, also belonged to the basal group, as the ancestor of some limes (*Citrus aurantifolia* [Christm.] Swingle) (Nicolosi et al., 2000; Froelicher et al., 2011; Curk et al., 2015; Wu et al., 2018). These four basic taxa led, through interspecific crossing, to the creation of the secondary species *Citrus sinensis* (L.) Osb. (sweet orange), *Citrus aurantium* L. (sour orange), *Citrus paradisi* Macf. (grapefruit), *Citrus limon* (L.) Burm. (lemon), and *Citrus aurantiifolia* (Christm.) Swing. (lime). Facultative apomixis limited the number of interspecific recombinations, and most of the genomes of modern citrus varieties are a mosaic of large fragments inherited from the ancestral taxa (Curk et al., 2016; Oueslati et al., 2017; Wu et al., 2018; Ahmed et al., 2019).

Much of the phenotypic diversity of edible citrus has resulted from the initial differentiation between the basic taxa (Barrett and Rhodes, 1976; Ollitrault et al., 2003), particularly for secondary metabolite contents such as carotenoids (Fanciullino et al., 2006) and furanocoumarins (Dugrand-Judek et al., 2015). Thus, the interspecific mosaic genome structure is a key component driving the ideotype of the secondary species and the phenotypes of modern varieties. Deciphering the interspecific admixture structures of the citrus germplasm and new hybrids is therefore essential for efficient utilization of citrus’s biodiversity in innovative breeding schemes (Curk et al., 2015; Ahmed et al., 2019), and developing molecular markers to diagnose the phylogenetic origin of the genes of the biosynthesis pathways for primary and secondary metabolites should open the way for targeted association studies and marker-assisted selection in sexual progeny.

Next generation sequencing (NGS) data dramatically improved our understandings of citrus domestication and revealed the phylogenomic structures of modern species and varieties (Wu et al., 2014; Wu et al., 2018). Whole genome sequencing (WGS) and genotyping by sequencing showed, in particular, that all edible modern mandarins resulted from introgressions of *C. maxima* genome fragments in a *C. reticulata* genomic background (Oueslati et al., 2017; Wu et al., 2018), suggesting a contribution by *C. maxima* during the mandarin-domestication process. Natural admixture of the *C. maxima* and *C. reticulata* gene pools also generated very important secondary species such as sour oranges, sweet oranges, and grapefruits (Wu et al., 2014; Wu et al., 2018). In combination with citrons, it contributed to the genesis of lemons (Curk et al., 2016; Wu et al., 2018; Ahmed et al., 2019). More recently, sexual-breeding programs have exploited the *C. maxima/C. reticulata* gene pools to develop the tangor (mandarin × sweet oranges) and tangelo (mandarin × grapefruit) horticultural groups. The identification of molecular markers not only differentiating *C. maxima* from *C. reticulata* gene haplotypes but also revealing intra-*C.maxima* or *C. reticulata* polymorphisms is therefore particularly interesting to optimize genetic studies and breeding within the *C. reticulata/C. maxima* admixture gene pool.

Single-nucleotide polymorphisms (SNPs) are codominant genetic markers with wide distribution throughout the genome, allowing the development of molecular taxonomic keys at the family or subfamily level (Brookes, 1999; Edwards and Batley, 2010). Nuclear SNP markers have been successfully developed and used in several studies on the phylogenetic relationships among citrus varieties (Ollitrault et al., 2012; Garcia-Lor et al., 2013; Curk et al., 2014; Wu et al., 2014; Curk et al., 2015; Curk et al., 2016; Oueslati et al., 2017; Wu et al., 2018). The recent genome-wide sequencing of citrus varieties (Wu et al., 2014; Wu et al., 2018) and genotyping by sequencing (GBS) studies (Oueslati et al., 2017; Ahmed et al., 2019) have drastically increased the availability and efficiency of SNPs for phylogenomic analyses. These studies have allowed the identification of numerous diagnostic SNPs (DSNPs) for the four ancestral taxa (Wu et al., 2018; Ahmed et al., 2019). The identification of SNPs from expressed sequence tags (Chen and Gmitter Jr, 2013) opens the way for further inferences about the relationships between specific gene polymorphisms and phenotypical characteristics.

Our work focuses on genes involved in specific metabolic pathways related to key traits of fruit quality: the carotenoid (involved not only in pulp and skin color but also in health properties), sugar (involved in the fruit’s flavor through the acidity/sugar balance), and chlorophyll (synthesis and degradation, as elements involved in the green-to-orange color transition during fruit maturation) genes. The objective was to identify and validate a set of SNPs differentiating ancestral *C. maxima* from *C. reticulata* haplotypes or identifying different haplotypes within *C. reticulata* and *C. maxima*. Gene haplotypes were established from publicly available resequencing data of varieties of the *C. reticulata/C. maxima* gene pool, taking advantage of the known parentage and the reference genome sequence of a haploid clementine (Wu et al., 2014). We successfully identified and developed SNP markers based on competitive PCR. We analyzed their transferability within the Citrus genus, identified the ancestral alleles and estimated the usefulness of the developed markers for tracing the ancestral haplotype in large germplasm collections and sexual recombining progenies issued from the *C. reticulata/C. maxima* admixture gene pool.

## Material and Methods

### Plant Material

Leaves from 92 accessions (**Supplementary Table 1**) of the *Citrus* genus and related genera were collected from the INRA/Cirad CRB-citrus collection of San Giuliano (Corsica, France). The Tanaka (Swingle and Reece, 1967) botanical classification for scientific names was adopted (**Supplementary Table 1**). The four ancestral taxa of the *Citrus* genus were represented by 38 accessions, comprising 22 mandarin (*C. reticulata*), 10 pummelo (*C. maxima*), 5 citron (*C. medica*), and 1 papeda (*C. micrantha*) accessions. The representatives of secondary citrus species included 21 other varieties: 2 clementines (*Citrus clementina;* one diploid and one haploid), 2 sour oranges (*C. aurantium*), 2 sweet oranges (*C. sinensis*), 3 grapefruits (*C. paradisi*), 10 lemons and limes (*C. aurantiifolia*, *Citrus bergamia*, *Citrus jambhiri*, *Citrus limetta*, *Citrus limettioides*, *C. limon*, *Citrus limonia*, and *Citrus meyeri*), 1 Combava (*Citrus hystrix*), and Nasnaran (*Citrus amblicarpa*). Twenty-six recent hybrid varieties within the C. reticulata/C. maxima gene pool (like tangelos, tangors, and orangelos) were also analyzed. Seven accessions of other genera within the Aurantioideae subfamily were added to test the transferability of the SNP markers and identify the ancestral allele. According to the subdivision proposed by Swingle and Reece (1967), one accession of the primitive citrus fruit group (*Severinia buxifolia*) was included, as were six accessions of the true citrus fruit trees group, which is closely related to the *Citrus* genus (*Poncirus*, *Clymenia, Eremocitrus, Microcitrus,* and *Fortunella*).

### DNA Extraction

Genomic DNA was extracted from leaf samples using the mixed alkyl trimethyl ammonium bromide (MATAB) methodology (Gawel and Jarret, 1991). The leaf samples were lyophilized and macerated with magnetic stainless spheres; then, 1 ml of extraction buffer (1.4 M NaCl, 100 mM Tris–HCl pH 8.0, 20 mM EDTA, 10 mM Na2SO3, 1% PEG 6000, 2% MATAB) preheated to 75°C was added. Each extract was homogenized by inversion, incubated for 30 min at 75°C, and kept at room temperature until cooling. Then, an equal volume of chloroform-isoamyl alcohol (24:1 v/v) was added to each extract and mixed by inversion. The tube was centrifuged at 7,000 g for 15 min, and the supernatant was precipitated at −20°C overnight after the addition of an equal volume of isopropanol. The DNA was isolated by centrifugation at 10,000 g for 10 min and resuspended in 100 µl of Milli-Q water. Genomic DNA concentration was normalized to 30 ng/µl for subsequent KASPar™ analysis.

### Search for *C. reticulata*/*C. maxima* Discriminant SNPs and Kaspar™ Marker Development

#### Search for SNPs From the Resequencing Data

**Figure 1:**
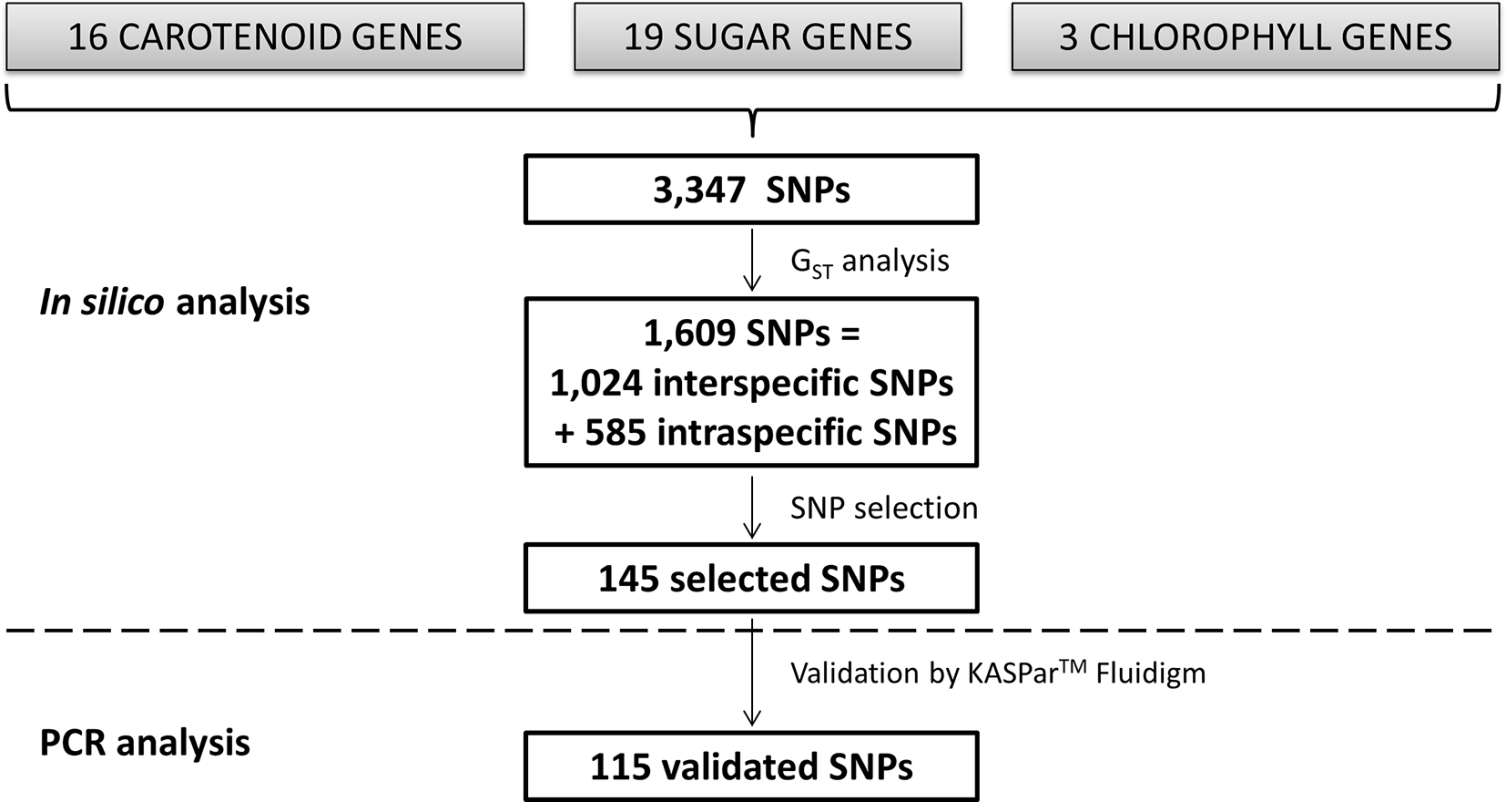
Scheme of the *in silico* analysis leading to SNP identification and KASPar^™^ analysis.

SNPs were identified by *in silico* analysis, as described in **Figure 1.** SNP mining was performed in specifically selected genes involved in determining citrus fruit quality: i) 16 carotenoid genes involved in pulp and skin color, ii) 19 genes involved in sugar biosynthesis pathways, and iii) three genes involved in chlorophyll metabolism (synthesis and degradation) (**Figure 1**; **Table 1**). Genes were selected from the annotation of the reference whole genome *C. clementina* v1.0, available at the Phytozome v11.0 database (https://phytozome.jgi.doe.gov/pz/portal.html). Gene sequences, structures (exons/introns and size), and positions in the genome were mined in the same database (**Table 1**). All genes were nuclear and located in the nine first scaffolds of the reference sequence corresponding to the nine citrus chromosomes. To distinguish the different genes from a multigene family, we added the number of the scaffold to the gene name plus a letter if several genesfrom a same family were located in a same scaffold (e.g., PDS_9a and PDS_9b are two phytoene desaturase genes located on scaffold 9). Available resequencing data of 10 citrus diploid varieties were used to identify SNPs within these genes, from *C. clementina* (Clementine Nules, SRX371962), 4 cultivars of *C. reticulata* (Willowleaf, SRX372685; Ponkan, SRX372665; Cleopatra, SRX2442472; and Nadorcott, SRX372687), 1 cultivar of *C. sinensis* (Ridge Pineapple,SRX372703), 1 cultivar of C. paradisi (Duncan; SRX2442478), 1 cultivar of *C. aurantium* (Seville, SRX372786), and 2 cultivars of *C. maxima* (Chandler, RX372688; and Acidless, SRX372702). Previous genomic data (Wu et al., 2014) have shown that Chandler and acidless pummelos are pure representatives of *C. maxima* and that Wu et al. (2018) concluded that the Cleopatra mandarin is almost a pure representative of C. reticulata, while the other varieties displayed *C. reticulata/C. maxima* admixture. The resequencing data were mapped onto the haploid clementine reference genome (Wu et al., 2014) using BWA-MEM, v0.7.12-r1039 (Li and Durbin, 2010), and variant calling was performed with Genome Analysis Toolkit Mckenna et al., 2010).

**Table 1:** List and characteristics of the genes used in this study. Chrm.: chromosome. R: C. reticulata. M: C. maxima.

	Gene								Number of SNPs			
	Code	ID	Chrm.	Beginning	End	Size (bp)	Number of introns	Number of exons	Total/ SNP	Interspecific RM (%)	Intraspecific	
											(%)	Type
Carotenoids	CCS_8	Ciclev10028245m	8	19259623	19261508	1,886	0	1	48	14 (29.1)	6 (12.5)	MM
	Sugars	GT_9	Ciclev10004221m	9	15981287	15989471	8,185	6	6	105	48 (45.7)	33 (31.4)	MM
		Clorophyll	PAO_8	Ciclev10028147m	8	21112804	21117215	4,412	6	7	159	19 (11.9)	13 (8.1)	RR (6) MM (7)
CRTISO_6	Ciclev10011230m	6	19578179	19583475	3,018	5	6	104	23 (22.1)	0 (0)	–
DXS_1	Ciclev10007595m	1	2200405	2205620	2,580	9	10	89	46 (51.7)	18 (20.2)	MM
DXS_7	Ciclev10024949m	7	5042508	5046278	2,676	9	10	72	23 (31.9)	15 (20.8)	RR
DXS_9	Ciclev10004432m	9	2353442	2357780	2,786	9	10	71	29 (40.8)	8 (11.26)	RR
HYB_9	Ciclev10005481m	9	29488658	29491181	2,524	6	7	55	17 (30.9)	4 (7.27)	RR
LCYb_9	Ciclev10004730m	9	22728310	22730086	1,777	1	2	27	16 (38.5)	8 (15.7)	MM
LCYe_1	Ciclev10008410m	1	10946316	10949617	3,302	6	7	39	21 (53.8)	0	–
NCED_2	Ciclev10014639m	2	35235517	35237892	2,376	0	1	44	11 (25.0)	4 (9.1)	RR
NCED_3	Ciclev10019364m	3	29351854	29354190	2,337	0	1	32	15 (46.8)	1 (3.1)	RR
NCED_9	Ciclev10006710m	9	10202439	10204217	1,779	0	1	50	8 (16.0)	21 (42.0)	RR (11) MM (10)
PDS_9a	Ciclev10005632m	9	17789634	17793258	3,625	6	7	54	24 (44.4)	9 (16.6)	MM
PDS_9b	Ciclev10007114m	9	17778571	17785482	6,912	5	6	143	81 (56.6)	16 (11.1)	MM
PSY_6	Ciclev10011841m	6	21390477	21396087	5,611	5	6	172	45 (26.1)	36 (20.9)	RR (17) MM (19)
ZEP_7	Ciclev10025089m	7	3222483	3228894	6,412	13	14	98	40 (40.8)	16 (16.3)	MM
Z-ISO_3	Ciclev10020648m	3	39692471	39696185	1,644	3	4	87	22 (25.2)	20 (22.9)	MM
INV_6	Ciclev10013701m	6	20333619	20338112	4,494	5	6	141	36 (25.5)	42 (29.7)	RR
INV_7a	Ciclev10025243m	7	6557251	6560551	3,301	3	4	80	14 (17.5)	14 (17.5)	RR
INV_7b	Ciclev10025259m	7	6561319	6564055	2,737	3	4	39	24 (61.5)	3 (7.7)	RR
INV_9	Ciclev10004465m	9	27282487	27287560	5,074	5	6	158	24 (15.2)	42 (26.5)	MM
SPP_2	Ciclev10015425m	2	645817	651088	5,272	6	7	69	41 (59.4)	6 (8.7)	MM
SPP_6	Ciclev10011822m	6	19586148	19590349	4,202	3	4	111	33 (29.7)	19 (17.1)	MM
SPS_1	Ciclev10007311m	1	25880824	25887976	7,153	6	7	149	68 (45.6)	19 (12.75)	MM
SPS_1a	Ciclev10007312m	1	1634245	1639542	5,298	6	7	130	1 (0.76)	77 (59.2)	MM
SPS_3	Ciclev10018655m	3	23001026	23009675	8,650	9	10	243	31 (12.7)	31 (12.7)	RR (7) MM (24)
SUSY_1a	Ciclev10010343m	1	24769195	24772566	3,372	10	11	67	21 (31.3)	18 (26.8)	RR
SUSY_1b	Ciclev10007483m	1	24814539	24820057	4,649	6	7	60	20 (33.3)	11 (18.3)	RR
SUSY_3	Ciclev10018889m	3	46011238	46017249	6,012	10	11	83	23 (27.7)	17 (20.5)	RR
SUSY_6	Ciclev10011062m	6	21404402	21408079	3,678	6	7	43	24 (55.8)	9 (20.9)	MM
SUSY_9	Ciclev10004341m	9	1499634	1505333	5,700	12	13	69	32 (46.3)	6 (8.7)	RR
SUT1_5	Ciclev10000828m	5	39525923	39529412	3,490	2	3	97	10 (10.3)	0 (0)	–
SUT2_4	Ciclev10030996m	4	23705523	23711365	5,843	9	10	105	38 (36.1)	16 (15.2)	RR
SUT4_5	Ciclev10000941m	5	35065343	35070910	5,568	4	4	75	27 (36)	16 (21.3)	RR
FEH_1	Ciclev10007827m	1	4119709	4122521	2,813	3	4	76	44 (57.8)	0 (0)	–
GDR_2	Ciclev10015206m	2	10231921	10233354	1,432	0	1	48	4 (8.3)	7 (14.6)	MM
GDR_3	Ciclev10020061m	3	7268419	7270557	2,139	1	2	55	7 (12.7)	4 (7.2)	MM
								Total	3,347	1,024	585	–

#### Haplotype Inference

The reference genome sequence was obtained from a haploid line of “Nules” clementine and therefore provided haplotype data for each gene. For each considered gene, the second haplotype of the diploid clementine was directly deduced through comparison of the diploid and haploid data. For the heterozygous “Willow leaf” mandarin and Ridge Pineapple sweet orange varieties, we took advantage of their kinship with the clementine (clementine is a direct hybrid “Willow leaf” mandarin × sweet orange; Ollitrault et al., 2012; Wu et al., 2014) to infer, for each gene, the two haplotypes of each variety. We first identified the clementine haplotype inherited from sweet orange and the one from Willow leaf mandarin. It was based on the incongruences of sequencing data with the two potential models (i.e., homozygosity of the considered parent for the alternative allele of the considered clementine haplotype). Comparing these haplotypes with each diploid parent genotype allowed the inference of the second haplotype. Moreover, sweet orange is one of the genitors of grapefruits (Wu et al., 2014; Oueslati et al., 2017; Wu et al., 2018); using the same approach, it was therefore possible to infer the two Duncan grapefruit haplotypes. For the other varieties (Nadorcott, Ponkan, and Cleopatra Mandarin; Chandler and acidless pummelo; sour orange) without direct parental relationship with previously haplotyped ones, haplotypes were inferred gene by gene from their resequencing data by statistical approach using the Genotype Visualization and Algorithmic Tool software (Davidovich et al., 2007) integrated in SNiPlay (http://sniplay.southgreen.fr/cgi-bin/home.cgi). The haplotype networks were established with Haplophyle software also integrated in SNiPlay. This analysis included the previously identified haplotypes and the additional diploid genotypes.

#### Search for *C. reticulata*/*C. maxima* Discriminant SNPs

Genetic relationships between the 10 citrus variety genotypes and the haplotypes of four of them were analyzed for each gene through factorial analysis with the DARwin software, v.6.0.014 (http://darwin.cirad.fr/;(Perrier and Jacquemoud-Collet, 2006), using the simple matching dissimilarity index. To identify discriminant SNPs between *C. maxima* and *C. reticulata*, the GST indice was estimated for each SNP considering *C. maxima* and *C. reticulata* populations constituting the corresponding haplotypes and pure diploid varieties. The analysis was performed on the allele frequency of each subpopulation (Ti and Tj) and of the whole population (Tot), as indicated in the formula below, where He is the genetic diversity within the population (He = 1 − Σpi2, where pi is the frequency of a the i allele in the considered population). GST ranged from 0 to 1, and the markers with GST = 1 were retained as discriminating between *C. reticulata* and *C. maxima*. The other SNPs revealed intraspecific diversity in *C. reticulata* or/and *C. maxima*.

$$G_{\mathrm{ST}}=\frac{He_{\mathrm{tot}}-\;\frac{\mathrm{He}{\;}_{\mathrm{Ti}}-\mathrm{He}{\;}_{\mathrm{Tj}}}{2}}{He_{\mathrm{tot}}}.$$

When clear differentiation between groups was observed at the intraspecific level (within *C. reticulata* or *C. maxima*), the same approach was applied to identify SNP markers revealing this intraspecific differentiation. Gene sequences (**Table 1**) from the 11 varieties indicated above were analyzed using the SNiplay online software (sniplay.southgreen.fr) to identify the SNP’s position in relation to the gene structure (exon/intron). SNPs located in the exons were preferred. A second filter criterion was the absence of additional SNPs at <25 bases. SNP locus-flanking sequences (50 bp upstream and downstream of each SNP) were provided to KBioscience for primer design and synthesis, as described by the KASPar™ technology (Cuppen, 2007). Among these sequences, most did not display additional SNPs (87) or only one (23) while seven and three displayed, respectively, two and three additional SNPs (**Supplementary Material, Table 4**). This information was taken into account for primers definition to limit bias in specific allele amplification.

### Kaspar™ Genotyping

KASPar™ genotyping and image analysis were performed on the Biomark™ Fluidigm^®^ platform available at Cirad (Montpellier, France). A 96 × 96 chip was used, allowing the genotyping of 96 individuals (genomic DNA at 30 ng/µl) with 96 markers in one PCR run, according to the KASPar™ technology’s instructions (Smith and Maughan, 2015). Briefly, two sources of fluorescence were used (FAM and ROX), each corresponding to one allele of the polymorphic gene. After PCR run, the chip was imaged, and at the end of the whole process, the software generated one graph for each KASPar^™^ marker × 96 genotypes. Detailed information about this genotyping method can be found in Cuppen, (2007). For each marker, homozygous individuals represented by one specific fluorescence source (FAM and ROX) were coded 1 and 0, respectively, while the heterozygous individuals amplifying both sources of fluorescence were coded 0.5.

### Diversity Analysis Based on Kaspar™ Genotyping

Principal component analysis (PCA) was computed using XLSTAT on the matrix of coded alleles “makers × varieties.” For varieties with haplotype inference, both diploid and haplotype genotypes were included. Diploid genotypes provided indicators of inter- or intraspecific structure location to help the phylogenetic assignation of diploid genotype data without haplotype inference. Heatmaps were generated using R v.3.1.2.

## Results

### *In Silico* Identification of SNPs and Segregation Analysis

Most of the considered genes are members of multigene families such as PDS, NCED, and DXS, in the case of carotenoids; GDR, in the case of the chlorophylls; or almost all the sugar genes (**Supplementary Table 2**). The selected genes were located in chromosome 1 (two carotenoid and five sugar genes), chromosome 2 (one carotenoid, one sugar, and one chlorophyll gene), chromosome 3 (two carotenoid, two sugar, and one chlorophyll genes), chromosome 4 (one sugar gene), chromosome 5 (two sugar genes), chromosome 6 (two carotenoid and three sugar genes), chromosome 7 (two carotenoid and two sugar genes), chromosome 8 (one carotenoid and one chlorophyll gene), and chromosome 9 (six carotenoid and three sugar genes) (**Table 1**). The carotenoid genes were 1,644–6,912 bp in length (Z-ISO_3 and PDS_9b, respectively); the sugar genes were 2,737–8,650 bp in length (INV_7b and SPS_3, respectively); and the chlorophyll genes were 1,432–4,412 bp in length (GDR_2 and PAO_8, respectively) (**Table 1**). They presented 0–13 introns (**Table 1**). A total of 3,347 SNPs were identified by *in silico* analysis from 38 genes related to carotenoid biosynthesis, sugar biosynthesis, and chlorophyll metabolism (**Figure 1**; **Table 1**). Each gene sequence contained 27–243 SNPs (LCYb_9 and SPS_3, respectively; **Table 1**).

**Figure 2:**
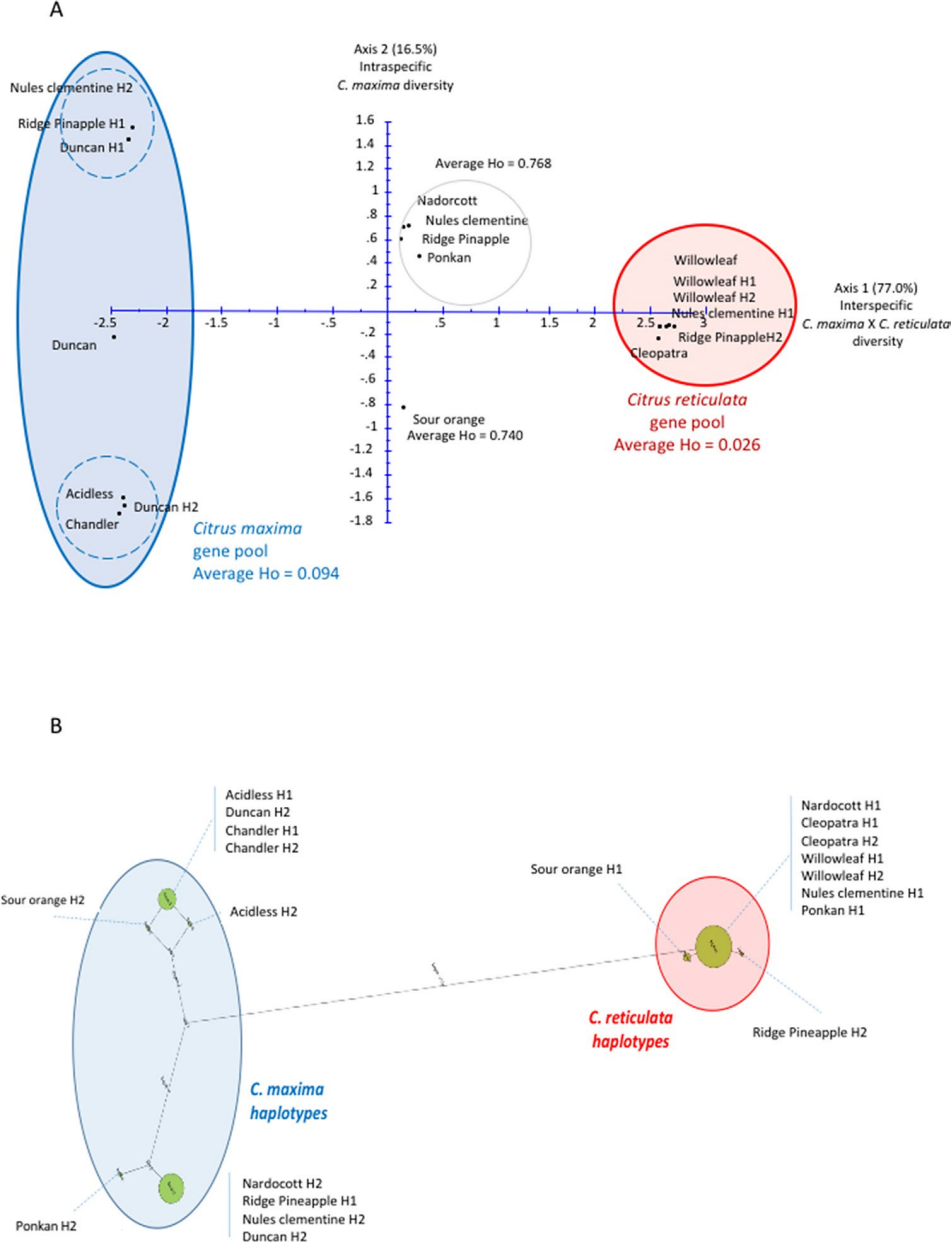
Factorial and haplotype analyses for the LCY-b gene. **(A)** Factorial analysis. H1 and H2 represent the haplotypes of the indicated corresponding variety. **(B)** Haplotype network. The size of the cirlces is proportional to haplotype frequency and the length of the lines is proportional to the number of mutation steps between genotypes and their proximal states. H1 and H2 represent the haplotypes of the indicated corresponding variety.

The phylogenetic origin of the haplotypes and the phylogenic constitution of the diploid genotypes were identified by factorial analysis. An example is provided for the LCYb_9 gene (**Table 1**; **Figure 2**), which displays a total of 27 SNPs. These SNPs clearly separate *C. maxima* and *C. reticulata* on axis 1 of the factorial analysis (**Figure 2A**) with 77% of the total variability. In addition, in axis 2, an intraspecific differentiation was observed within the pummelo group (**Figure 2A**); it accounts for 16.5% of the total variability. For this gene, the Willow leaf (both haplotypes) and Cleopatra mandarins, one of the Clementine Nules haplotypes (Clementine H1), and one sweet orange Ridge Pineapple haplotype (Ridge PineappleH2) were grouped, and they displayed low intraspecific polymorphisms (**Figure 2**); they were considered representatives of *C. reticulata* for the GST analysis of the SNPs of the LCYb_9 gene. *C. maxima* was represented by two haplotype groups: i) ClementineH2, Ridge PineappleH1, and DuncanH1 on the left and ii) DuncanH2, acidless and Chandler pummelos on the right. The *C. maxima* representative group showed greater polymorphism than the *C. reticulata* group, and a discriminant marker for the two *C. maxima* haplotype groups was selected. Interestingly, Nadorcott and Ponkan constituted a cluster with clementine and sweet orange diploid genotypes in an intermediary position between *C. maxima* and *C. reticulata* gene pools and presented high heterozygosity values (0.768) in average in comparison with the low 0.094 and 0.026 values, respectively for *C. maxima* and *C. reticulata* gene pools. These highly heterozygous varieties can therefore be considered to be in interspecific *C. maxima/C. reticulata* heterozygosity for the LCYb_9 gene. The same conclusion can be applied to sour orange with 0.740 heterozygosity value and intermediary position between the two ancestral gene pools.

This phylogenetic origin study from factorial analysis was fully validated from the haplotype network analysis (**Figure 2B**). Two haplotype pools corresponding to *C. maxima* and *C. reticulata* were clearly differentiated. The *C. maxima* haplotype of Nadorcott was shared with one haplotype of sweet orange, clementine, and grapefruit, and the one from Ponkan was very closely related. The *C. maxima* haplotype from sour orange was closely related to those of the two pummelos. Very little haplotypic diversity was observed for the *C. reticulata* side, and the *C. reticulata* haplotypes of Ponkan and Nadorcott were shared with Willow leaf and Cleopatra mandarins.

Factorial analysis was performed for each gene, which revealed clear differentiation between *C. maxima* and *C. reticulata* clusters and, therefore, the identity of the haplotype origin and diploid phylogenetic structure of the diploid varieties. Haplotype network analysis also confirmed the important differentiation between *C. reticulata* and *C. maxima* gene pools (illustrated in **Supplementary Data 1** for carotenoid biosynthesis pathway genes) and confirmed the conclusions of factorial analysis for all analyzed genes. However, considering the small number of varieties used for statistical haplotypes inference, and therefore potential bias, we preferred not to use the haplotypes statistically deduced from interspecific heterozygous varieties (such as Ponkan, Nadorcott, and sour orange for LCYb_9 gene) for GST analysis. For each gene, *C. reticulata* haplotypes plus pure *C. reticulata* diploid mandarins, on the one hand, and *C. maxima* haplotypes plus pure *C. maxima* pummelo, on the other hand, were selected to compute *C. reticulata* and *C. maxima* allelic frequencies, respectively, and to estimate GST for each SNP of the considered gene. For some genes, as previously mentioned for LCYb_9, we observed additional intraspecific *C. maxima* or *C. reticulata* polymorphisms (data not shown).

**Figure 3:**
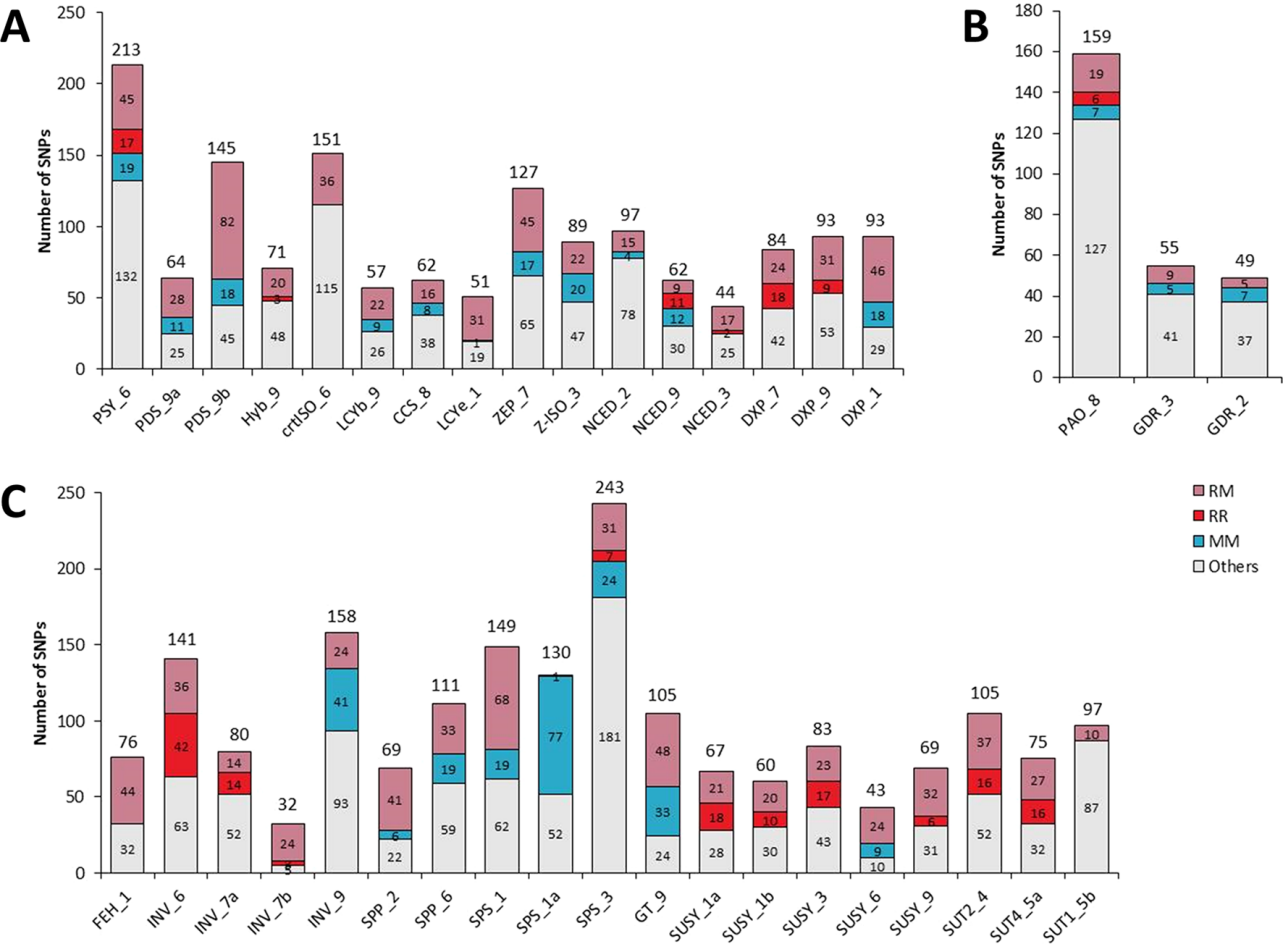
Classification of SNPs. **(A)** Genes related to the carotenoid metabolic pathway. **(B)** Genes related to the sugar metabolic pathway. **(C)** Genes related to the chlorophyll metabolism. RM indicates SNPs segregating C. reticulata from C. maxima (interspecific segregation). RR indicates SNPs allowing intraspecific segregation of C. reticulata. MM indicates SNPs allowing intraspecific segregation of C. maxima. The Others category corresponds to SNPs that allowed segregation different from RM, RR, or MM. The total number of SNPs is indicated at the top of each column.

From the 3,347 SNPs, 1,024 were potentially discriminant between *C. maxima* and *C. reticulata*, while 585 were useful for discriminating groups within *C. maxima* or *C. reticulata* (**Figure 1**; **Table 1**). Among the carotenoid genes, PDS_9b, DXS_1, PSY_6, and ZEP_7 contained the highest numbers of interspecific SNPs (81, 46, 45, and 40, respectively; **Table 1**; **Figure 3A**). The sugar genes with the highest numbers of interspecific SNPs were SPS_1, GT_9, FEH_1, and SPP_2, with 68, 48, 44, and 41 SNPs, respectively (**Table 1**; **Figure 3C**). Seven carotenoid genes (DXS_7, DXS_9, HYB_9, NCED_2, NCED_9, NCED_3, and PSY_6) and 10 sugar genes (INV_6, INV_7a, INV_7b, SPS_3, SUSY_1a, SUSY_1b, SUSY_3, SUSY_9, SUT2_4, and SUT4-5) contained SNPs involved in C. reticulata diversity (**Table 1**; **Figure 3**). Additionally, nine carotenoid (CCS_8, DXS_1, LCYb_9, NCED_9, PDS_9a,PDS_9b, PSY_6, ZEP_7, and Z-ISO_3) and eight sugar (GT_9,INV_9, SPP_2, SPP_6, SPS_1, SPS_1a, SPS_3, and SUSY_6) genes displayed *C. maxima* polymorphisms (**Table 1**; **Figures 3A, C**). The chlorophyll genes presented few SNPs discriminating *C. reticulata* from *C. maxima* (**Table 1**; **Figure 3B**). Interestingly, some genes presented polymorphic SNPs within both *C. reticulata* and *C. maxima* (NCED_2, PSY_6, SPS_3, and PAO_8 ; **Figure 3**).

### Kaspar™ Analysis for Marker Validation and Inferences on the Genetic Diversity of Citrus

For marker validation by KASParTM, 145 potentially interesting SNPs for inter- and intraspecific studies within the *C. reticulata/C. maxima* gene pool were selected (**Figure 1**); for each gene, at least two interspecific and one intraspecific SNPs were chosen. The validation was performed on 92 accessions of citrus (**Supplementary Table 1**), and 115 SNPs of the 145 (79%) allowed efficient genotyping (**Figure 1**; **Supplementary Table 4**). Among them, 79 were initially selected for *C. reticulata/C. maxima* differentiation, and 18 and 20 were selected for intra-*C. reticulata* and *C. maxima* diversity, respectively. The transferability of these markers mined from the *C. reticulata/C. maxima* gene pool was very good, with only 0.87% of missing data in the whole dataset. However, this ranged from 0.74% for the *Citrus* representatives to 1.24% for the other true citrus genera (*Poncirus*, *Fortunella, Clymenia, Eremocitrus,* and *Microcitrus*) and 3.48% in S. buxifolia (the outgroup of the true citrus).

**Figure 4:**
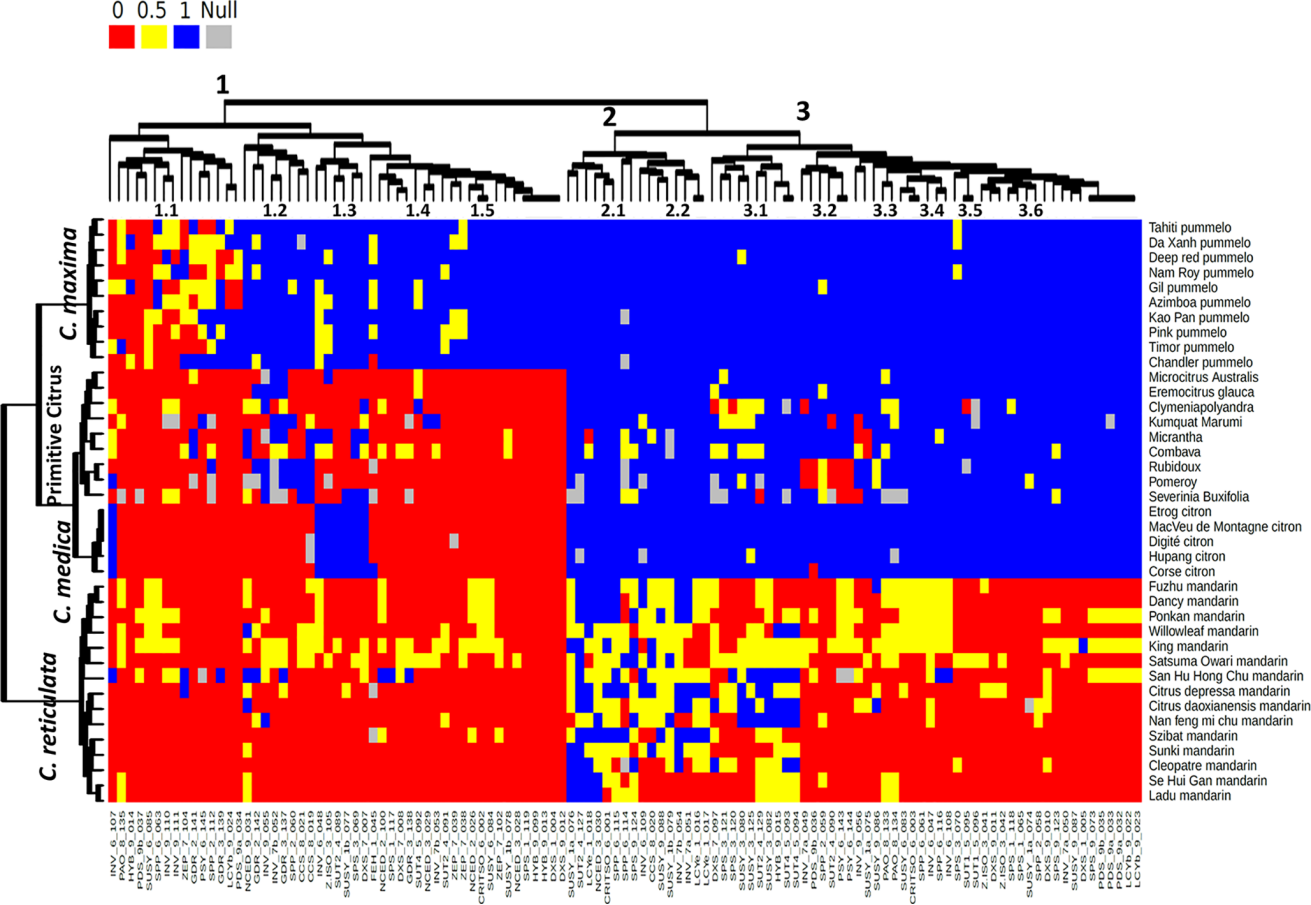
Heatmap of 67 genotyped citrus varieties along with 115 SNP markers. The red, blue, and yellow colors correspond to SNP homozygous for the C. reticulata allele (0), homozygous for C. maxima allele (1), and heterozygous (0.5), respectively. Gray color indicates null alleles (undefined origin).

In order to analyze the link between specific alleles and citrus groups and to infer the ancestral allele for each locus, a heatmap analysis was performed on a dataset including the representatives of the four ancestral taxa of the *Citrus* genus and of the other genera, specifically of 15 mandarin, 10 pummelo, 5 citron (*C. medica*), 2 papedas (wild representatives of the *Citrus genus*), and 7 other citrus genera, using the 115 functional SNPs (**Figure 4**). Three main clusters were observed for the accessions: one cluster for mandarins, one cluster for pummelos, and one cluster joining the others accessions of the true citrus and *S. buxifolia*. This third cluster joining highly differentiated species (Wu et al., 2018; Carbonell-Caballero et al., 2015) confirmed that most of the selected polymorphisms were specifically of *C. reticulata* or *C. maxima* and were fixed for the ancestral allele in the other species. In the third cluster, a subcluster joined all the *C. medica* representatives displaying very few polymorphisms and heterozygosity. The heatmap showed three clusters for the SNP categorization (**Figure 4**). Cluster 1 included markers with specific alleles in *C. maxima*, differentiating it from most of the other species. It can be supposed that the ancestral allele is the one shared by the other species. The markers of subcluster 1.1 were polymorphic within mandarins and pummelos and therefore did not allow differentiation of *C. maxima* and *C. reticulata* accessions. Subcluster 1.2 joined SNPs displaying one allele shared by *C. medica* and *C. maxima* and not present in the other accessions. They should be useful for differentiating *C. maxima/C. reticulata* haplotypes when working in the *C. maxima/C. reticulata* admixture gene pool. Subclusters 1.3 and 1.4 mainly corresponded to polymorphic markers within pummelos that are not useful for *C. maxima/C. reticulata* differentiation. Subcluster 1.5 joined the markers with all pummelo cultivars fixed for a specific allele, which probably occurred after the separation of *C. maxima* from the other clades. Cluster 2 contained markers (e.g., GDR_2_141, INV_6_107, ZEP_7_104, HYB_9_014, LCYe_1_018, NCED_3_030, SPP_6_063, and SUSY_6_085) revealing mainly intraspecific diversity without complete differentiation between *C. reticulata* and *C. maxima*. Subcluster 2.1 displayed polymorphisms in most of the groups (excepted C. medica), including the related genera. It probably corresponded to very old polymorphisms inherited in the different phylogenetic clades. Cluster 2.2 mainly corresponded to polymorphisms only present in mandarins, but they were not fixed in this group. It includes LCYe_1_016 and LCYe_1_017, identified as discriminants between *C. reticulata* and *C. maxima* in our *in silico* study with a very limited genotypic panel. These two markers are therefore not validated as interspecific markers but rather as *C. reticulata* polymorphisms. The corresponding mutations probably occurred in *C. reticulata* after this taxon’s separation from the other *Citrus* clades. Cluster 3 joined the markers differentiating *C. reticulata* and *C. maxima*. For subclusters 3.1, 3.2, 3.3, 3.4, and 3.5, polymorphisms were revealed between and within the genera of the true citrus, suggesting old polymorphisms inherited in the different clades except *C. maxima* for most of the considered SNPs. *C. medica* cultivars share the major allele of *C. reticulata* and *C. maxima* in subclusters 3.1 and 3.2, respectively. Interestingly, in subcluster 3.3, poncirus and kumquat share the major allele of mandarins for the Psy markers. The 26 markers of subcluster 3.3 shared similar patterns with most of the accessions, except mandarins fixed for the same allele, while mandarins are mainly fixed for the other allele. These SNPs probably resulted from mutation in the *C. reticulata* clade after its separation from the other clades (**Figure 4**).

**Figure 5:**
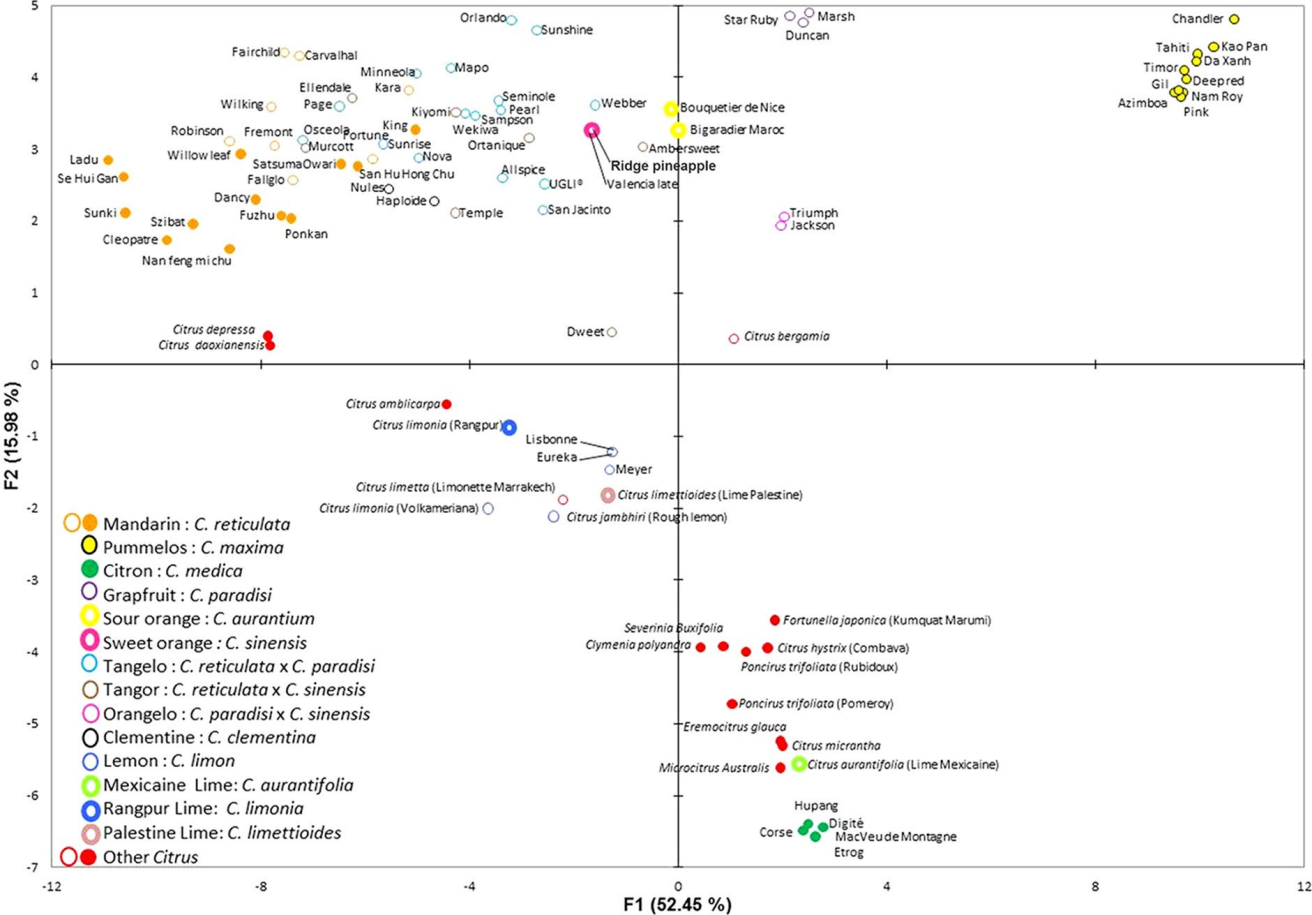
PCA of 92 citrus varieties using 103 nonredundant SNPs.

A PCA was performed on the 92 citrus varieties (**Figure 5**). About 69% of all diversity is represented in axes 1 and 2 (**Figure 5**). Axis 1 of the PCA mainly separated the mandarins (*C. reticulata*) from the pummelos (*C. maxima*) and also the citrons (*C. medica*), while axis 2 separated the pummelos from the citrons (**Figure 5**). In axis 1, other species associated with the *C. reticulata/C. maxima* gene pool such as *C. sinensis* (orange), *C. aurantium*, and *C. paradisi* (grapefruit), as well as their hybrids, such as tangelos (*C. reticulata × C. maxima*), tangors (*C. tangerina × C. sinensis*), orangelos (*C. paradisi × C. sinensis*), and clementines (*C. reticulata × C. sinensis*) were logically found in an intermediate position between mandarins and pummelos, closely linked to their relative *C. reticulata/C. maxima* constitution, as estimated by the gene-by-gene phylogenetic origin study (**Supplementary Material, Table 5**). Axis 2 isolated the citron population (*C. medica*) and several other ancestral genera and species of citrus such as Microcitrus australis, *C. aurantiifolia*, *C. micrantha*, *Eremocitrus glauca, Poncirus trifoliata, C. hystrix, Fortunella japonica, Clymenia polyandra,* and *S. buxifolia*. Lemon and lime varieties resulting from interspecific hybridization (*C. limon, C. jambhiri, C. limettioides, C. limonia, and C. limetta)* were located in intermediate positions between the previous group and the mandarin one. *C. bergamia* had a central position between the three main groups. The grouping of citron, papedas, and the other genera confirmed that most of the SNPs were specific to *C. maxima/C. reticulata* differentiation or intraspecific variability within *C. reticulata or C. maxima*. Interestingly, there is a remaining polymorphism allowing the differentiation of these species/genera. It corresponds to the SNPs identified by the heatmap study as being more ancient than the separation of these different species/genera.

Remarkably, from the 115 SNP markers tested, 23 were redundant and could be reduced to seven unique segregations (**Supplementary Table 3**). Some of these redundant SNPs were located in the same gene (LCYe_1_016/LCYe_1_017, SUT4_5_093/SUT4_5_094, PSY_6_143/PSY_6_144, HYB_9_099/HYB_9_013, LCYb_9_022/LCYb_9_023, and PDS_9a _032/PDS_9a _033) and in different genes located in the same chromosome (DXS_1_004/SPS_1b _119 in chromosome 1, CRITSO_6_003/SPP_6_061 and CRITSO_6_002/SUSY_6_084 in chromosome 6, and GT_9_073/PDS_9b _035/PDS_9a _032/LCYb_9_022 in chromosome 9) (**Supplementary Table 3**).

### Phylogenetic Origin of the Primary and Secondary Metabolic Pathway Genes in Citrus Germplasm

The main objective of this work was to develop diagnostic markers of the haplotypes inherited from *C. maxima and C. reticulata* for genes involving the carotenoid, the sugar biosynthesis pathway, and chlorophyll synthesis and degradation. The analysis of 92 citrus accessions validated 57 interspecific diagnostic markers of 13, 14, and 2 genes for the carotenoid, sugar, and chlorophyll pathways, respectively (**Supplementary Table 5**). Three, 25, and 1 of these genes had one, two, and four interspecific DSNP validated markers, respectively. Within the *C. reticulata/C. maxima* gene pool, these diagnostic markers can be used directly to trace the phylogenetic origin of the corresponding genes. For varieties with more complex origins involving *C. medica* as a direct parent, in addition to *C. reticulata and C. maxima, C. maxima,* or *C. reticulata* contribution, can be inferred when taking into account their phylogenetic origin.

**Figure 6:**
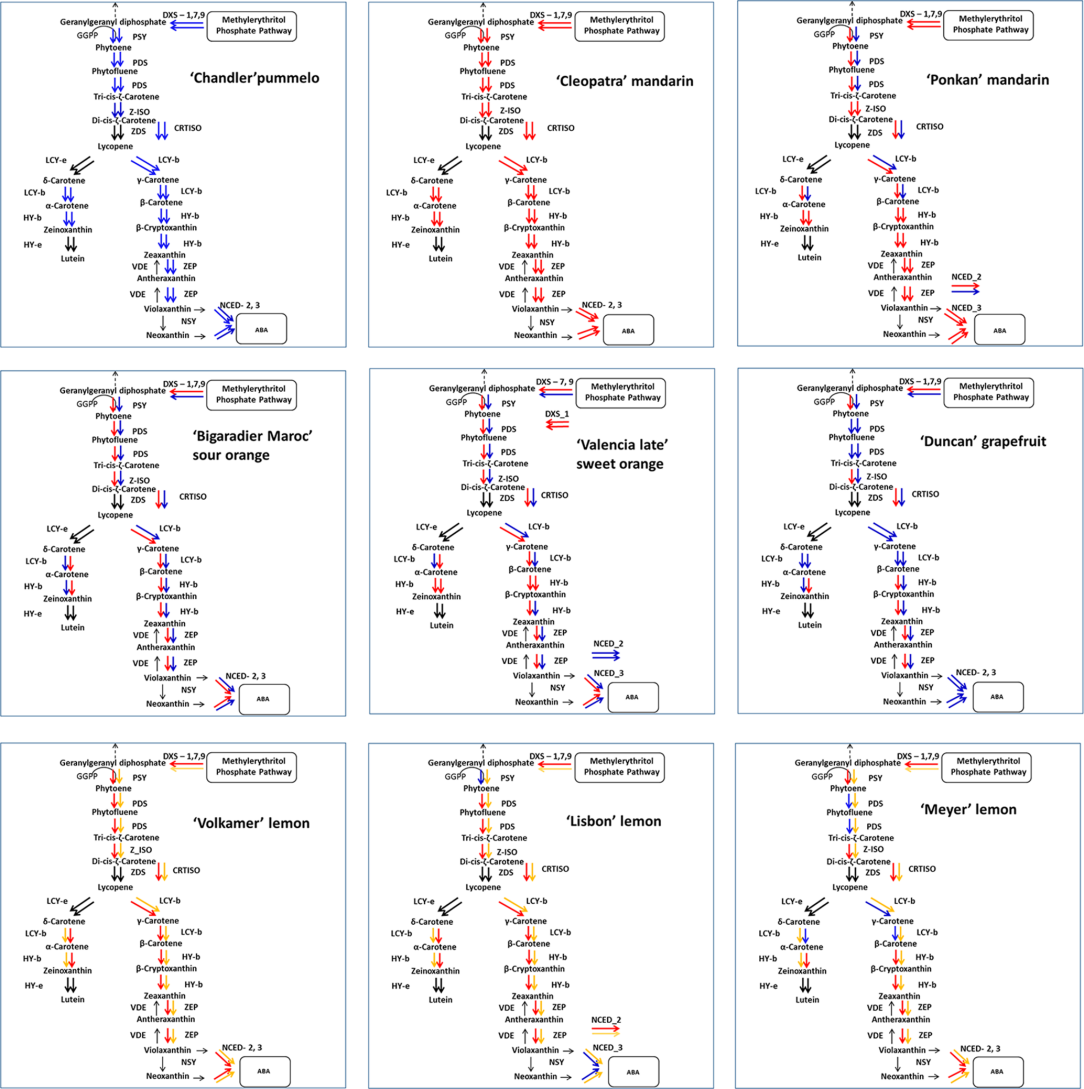
Allelic origins of the carotenoid pathway genes. The red and blue arrows indicate *C. reticulata* and *C. maxima alleles*, respectively. The yellow arrows indicate the *C. medica allele*. The black arrows correspond to alleles with undefined origin.

Examples are proposed in **Figure 6** for the carotenoid biosynthesis pathway. The figure showing this pathway was adapted from Fanciullino et al. (2006), with the addition of the Z-ISO gene, which mediates the production of 9,9’-di-cis-z-carotene (the substrate for ZDS) from 9,15,9’-tri-cis-z-carotene (Chen et al., 2010). As expected, the Cleopatra mandarin and Chandler pummelo, representative of *C. reticulata* and *C. maxima,* respectively (Oueslati et al., 2017; Wu et al., 2018), displayed specific homozygosity with contrasting alleles for all genes of the carotenoid metabolic pathway. However, several edible mandarins displayed some steps of the biosynthesis pathways with *C. maxima* alleles (**Figure 6** shows the example of Ponkan, while **Supplementary Table 5** shows all varieties), as already revealed from PCA analysis of the LCY-b gene’s haplotypic and genotypic data for Ponkan and Nadorcott (**Figure 2**). The secondary species *C. aurantium* (sour oranges) displayed interspecific *C. maxima/C. reticulata* heterozygosity for all characterized genes of the carotenoid pathway (**Figure 6**), while DXS_1 and HY-b were in *C. reticulata* homozygosity and NCED_2 in *C. maxima* homozygosity for the two sweet oranges (C. sinensis) analyzed. *C. paradisi* (three grapefruits analyzed) displayed a pattern of *C. maxima/C. reticulata* heterozygosity for DXS, PSY, Z-ISO, CRTISO, HY-b, and ZEP, and *C. maxima* homozygosity for PDS, LCY-b, and NCED. For acid citrus species involving citron (*C. medica*) as one direct parent in addition to the *C. maxima/C. reticulata* gene pool, we took advantage of the complete fixation of citrons for one allele, for 113 markers over the 115 analyzed, to infer the allele inherited from the second parent. The approach was validated with Volkamer lemon, which displayed complete *C. reticulata/C. medica,* in perfect agreement with its origin by direct cross between *C. reticulata* and *C. medica* (Curk et al., 2016; Ahmed et al., 2019). The patterns for the Lisbon and Meyer lemons were more complex. Both included *C. maxima/C. medica* and *C. reticulata/C. medica* heterozygosities but at different steps of the biosynthesis pathway. **Supplementary Table 5** provides the interspecific phylogenetic constitutions inferred for the 29 marked genes, for the varieties of the *C. reticulata/C. maxima* gene pool as well as the varieties with more complex origin where the segregation of *C. reticulata/C. maxima* haplotypes could be traced. For genes with two to four interspecific DSNPs, the conclusions were the same for the different markers of the same gene in 97% of cases (discrepancies were considered unidentified origins).

The same analysis was performed for 14 and two genes of the sugar and chlorophyll pathways, respectively (**Supplementary Material, Table 5**). No introgression was observed in pummelos. For mandarins, no introgression was found for two sugar pathway genes (SPP_2 and SPS_1). For the 12 remaining sugar genes, eight mandarins were not introgressed (*Citrus depressa*, Nan feng mi chu, San Hu Hong Chu, Szibat, Cleopatra, Ladu, Sunki, and Se Hui Gan). The other mandarins displayed between 1 (*Citrus daoxianensis*) to 9 (King) of the 14 sugar genes in *C. reticulata/C. maxima* heterozygosity. For the two chlorophyll genes, the level of C. maxima introgression in mandarins was high (28.4 %) with 8 and 13 varieties out of 22 in interspecific heterozygosity, respectively, for GDR_3 and PAO_8, and even 1 variety in *C. maxima* homozygosity for each gene. Sweet orange and sour oranges were in full interspecific *C. reticulata/C. maxima* heterozygosity for all analyzed chlorophyll and sugar genes, while grapefruits displayed three genes in *C. maxima* homozygosity (GRD_3, SPS_9 and SUT1_5) and the others in *C. reticulata/C. maxima* heterozygosity. Tangelos and tangors displayed a segregating pattern of *C. reticulata* homozygosity, *C. maxima* homozygosity, and *C. reticulata/C. maxima* heterozygosity. The Mediterranean Lisbon and Eureka lemons displayed four genes in *C. maxima/C. medica* heterozygosity and the others in *C. reticulata/C. medica* heterozygosity. As for carotenoid, the 2 chlorophyll and 14 sugar genes analyzed were fully heterozygous *C. reticulata/C. medica* in Rangpur lime and Volkamer lemon.

## Discussion

SNP genotyping has become a powerful tool for phylogenetic study and applications in breeding programs. Indeed, SNPs cover the whole genome, and in addition to being codominant genetic markers, they can reveal the functional variability of specific gene families or metabolic pathways when present in expressed sequence tag or coding genome regions (Chen and Gmitter Jr, 2013). A comparative phylogenetic analysis performed using SNPs, indels, and simple sequence repeat markers showed that SNPs were more useful for evaluating genetic variations among Citrus genera and intertaxon differences (Garcia-Lor et al., 2013). With recent genome-sequencing data (Wu et al., 2014; Wu et al., 2018) and GBS analysis (Oueslati et al., 2017; Ahmed et al., 2019), it was possible to perform deep analyses and to select diagnostic SNPs of the different ancestral citrus taxa. In this study, publicly available resequencing data of 10 modern varieties belonging to the *C. reticulata/C. maxima* gene pool were used to mine SNPs and to infer haplotypic gene sequences for species and varieties having a parental relationship with clementine for which haplotypic data were available (Wu et al., 2014). This is one of the few works focusing on SNPs related to specific metabolic pathways associated with fruit quality. The 38 analyzed genes covered 154.7 kb, and 3,347 SNPs were identified. The global SNP average rate was 21.6 SNPs/kb. This value was logically lower than those found by Curk et al. (2015) (36.7 SNPs/kb) and Garcia Lor et al. (2013) (52.9 SNPs/kb), respectively working within the whole Citrus genus and the true citruses, including the *Citrus,* Poncirus, Fortunella, Microcitrus, and Eremocitrus genera. From these 3,347 SNPs, 1,024 were potentially discriminant between *C. maxima* and *C. reticulata*, while 585 were useful for discriminating among subgroups within *C. maxima* or C. reticulata. After selection and validation by KASParTM methodology, a diagnostic set of 115 effective SNPs was obtained. Some of these markers presented redundant diagnostic patterns in the 92 accessions under study but may be still informative considering the associated gene function (e.g., SNPs located in genes from different metabolic pathways but presenting the same segregation pattern). The redundant markers could also reveal regions with strong genetic linkages. It is, for example, the case for redundant markers of the LG9 where very limited recombination was observed in a very wide genomic centromeric and pericentromeric area (Wu et al., 2014). All SNP markers mined within the *C. maxima/C. reticulata* gene pool were tested in several other true citrus species and genera-representative accessions. Very good transferability among all *Citrus* species and the true citrus genera was observed, with <0.9% of missing data, in agreement with previous works with the same allele-competitive PCR method (Garcia-Lor et al., 2013). The related genera of the true citruses were grouped and were close to the *C. medica* accessions for the used markers, while previous nuclear and chloroplatic phylogenomic data from WGS (Carbonell-Caballero et al., 2015; Wu et al., 2018) testified to the important genetic divergence between these taxa. This apparent similarity between very divergent taxa clearly illustrates bias due to the very partial representativeness of true citrus by the *C. maxima/C. reticulata* discovery panel. Such bias was discussed in previous works, based on SNPs mined from sequencing data of only one heterozygous genotype (Clark et al., 2005; Bradbury et al., 2011; Ollitrault et al., 2012). This grouping also confirms that, as expected, most of the selected SNPs are specific to *C. maxima/C. reticulata* differentiation or to the intraspecific variability within *C. reticulata* or *C. maxima*. The heatmap analysis also allowed to identify the ancestral alleles of the different SNPs and the clade where specific mutations occurred in case of *C. reticulata/C. maxima* diagnostic markers. For the modern varieties resulting from the *C. reticulata/C. maxima* gene pool (mandarins, pummelos, grapefruits, sweet oranges, sour oranges, tangors, tangelos, and orangelos), these markers were therefore powerful enough to identify the genes inherited from *C. reticulata* and *C. maxima* along the carotenoid, sugar, and chlorophyll pathways. Such pathways are involved in citrus fruit development and ripening, and several works highlighted the different possible mechanisms regulating them, such as retrotransposons (Butelli et al., 2012), miRNA (Wu et al., 2016), histone methylation (Xu et al., 2015), and/or transcriptional control through specific transcription factors (Zhu et al., 2017; Terol et al., 2019). However, the allele origin could also be considered in the regulation mechanisms leading to the phenotypic variation, as observed in other plant species during their development, and for some specific pathways such as sugar metabolism (Song et al., 2013; Albert et al., 2018). In these works, exonic SNPs allowed the identification of conserved unbalanced allelic expression between parents and F1 hybrids as signature of parental cis-regulatory divergences (Song et al., 2013; Albert et al., 2018). Here, we believe that the citrus carotenoid and sugar metabolic pathways could be regulated also through allelic variation inherited from *C. reticulata or C. maxima*.

The results provided by this phylogenetic inheritance analysis along the biosynthesis pathway were in total accordance with the ones from factorial analysis. For secondary species such as sweet and sour oranges and grapefruits, the contributions of *C. maxima* and *C. reticulata* estimated by gene-by-gene phylogenetic origin analysis were very close to the one estimated by genome-wide analysis (Oueslati et al., 2017; Wu et al., 2018). The C. maxima introgression, observed at whole genome level, in cultivated mandarin was also revealed by the species diagnostic SNPs for some of the considered carotenoid, chlorophyll, and sugar genes. Many molecular marker and genomic studies have documented the genetic relationship of secondary species with the citrus basic taxa (Barkley et al., 2006; Garcia-Lor et al., 2012; Ollitrault et al., 2012; Garcia-Lor et al., 2013; Curk et al., 2014; Curk et al., 2015; Curk et al., 2016; Wu et al., 2018; Ahmed et al., 2019). Our study, focused on carotenoid, sugar, and chlorophyll biosynthesis pathway genes, confirmed that C. aurantium (sour orange) resulted from a direct hybridization between *C. reticulata* and *C. maxima*, while *C. sinensis* (sweet orange) had a more complex history, with *C. reticulata* homozygosity for several loci resulting in a higher contribution of *C. reticulata* than *C. maxima*. The phylogenetic inheritance patterns of grapefruits, pummelo, and sweet orange genes and the intermediary position of grapefruits between sweet oranges and pummelos in the PCA analysis agree with an origin of grapefruit as a hybrid of sweet orange and pummelo (Ollitrault et al., 2012; Curk et al., 2015). The positions of tangors and tangelos on the factorial analysis between *C. maxima* and *C. reticulata* were in full agreement with the direct inference of phylogenomic constitution from DSNPs. Interestingly, despite the development of markers from a *C. maxima/C. reticulata* discovery panel, the selected SNPs could distinguish a third cluster in the factorial analysis, constituted by the other ancestral taxa of cultivated Citrus (*C. medica* and *C. micrantha*) and the other genera of the true citrus plus S. buxifolia. This third cluster included germplasm, characterized by ancestral alleles for the selected markers. The secondary species implying *C. medica* as one parent were positioned in agreement with the conclusions of previous molecular studies. For example, the Rangpur lime and Volkamer and Rough lemons supposed to result from direct hybridization between *C. reticulata* and *C. maxima* (Curk et al., 2016, Wu et al., 2018) were intermediate between the citron and mandarin clusters. Lemons (C. limon) were proven to result from hybridization between *C. aurantium* and *C. medica* (Nicolosi et al., 2000; Curk et al., 2016; Wu et al., 2018; Ahmed et al., 2019). They displayed a predominant inheritance of *C. reticulata* alleles from *C. aurantium* for the carotenoid, sugar, and chlorophyll pathways. The diagnostic markers of *C. maxima/C. reticulata* differentiation can be applied in large germplasm collections and hybrid populations involving these two species to trace the *C. reticulata* and *C. maxima* haplotypes. This will pave the way for targeted genetic association studies based on ancestral haplotypes (Bardel et al., 2009; De Roos et al., 2011; Zhang et al., 2012).

## Conclusion

Publicly available resequencing data of 10 modern varieties belonging to the *C. reticulata/C. maxima* gene pool were used to mine SNPs and infer haplotypic gene sequences related to metabolic pathways associated with fruit quality (carotenoid, chlorophyll, and sugars). Among the 3,347 SNPs identified, from a total of 154.7 kb of DNA sequences, we selected and validated a set of 115 SNP markers based on allele-competitive PCR. These SNPs were selected to differentiate *C. maxima* from *C. reticulata* and to identify intraspecific polymorphisms within *C. reticulata* and *C. maxima*. Their transferability among all *Citrus* species and the true citrus genera was very good, with only 0.87% of data missing. They revealed a genetic organization of the Citrus species in agreement with the previous hypothesis on citrus’s evolution and domestication. We identified the ancestral alleles of the SNPs and validated the usefulness of the developed markers for tracing the ancestral haplotype in large germplasm collections and sexually recombined progeny issued from the *C. reticulata/C. maxima* admixture gene pool. These markers will pave the way for targeted association studies based on ancestral haplotypes.

## Data Availability

All data generated or analyzed during the current study are included in this published article and its supplementary information files.

## Author Contributions

PO and FM conceived the experiment, MS conducted all of the experiments, and MFBA identified SNPs from sugar genes. FO contributed to the KASParTM analysis. RR and DG assisted MS during the KASParTM experiments. ES conducted the heatmap analysis. FL provided the plant material and contributed to the molecular analysis. MS, DG, FM, and PO analyzed the data and wrote the manuscript. AG, FL, and FM were responsible for the financial support of the research. FM supervised MP. FM, PO, FL, and DG advised MS during the different experimental steps at UESC, Cirad and INRA.

## Funding

MS and MP were funded by the Conselho Nacional de Desenvolvimento Científico e Tecnológico (CNPq). ES was funded by the Coordenação de Aperfeiçoamento Pessoal de Nível Superior (CAPES). This research was supported by CNPq (Pesquisador Visitante Especial call, the CAPES/Agropolis program, and the Innov’Agrumes Feder project. FM and AG received a Productivity Grant from CNPq (PQ). This work was made in the frame of the International Consortium in Advanced Biology (CIBA).

## Conflict of Interest Statement

The authors declare that the research was conducted in the absence of any commercial or financial relationships that could be construed as a potential conflict of interest.

## Abbreviations

NGS, next generation sequencing; SNP, single-nucleotide polymorphism; WGS, whole genome sequencing.
