# Supplementary material for: Phylogenetic Origin of Primary and Secondary Metabolic Pathway Genes Revealed by C. maxima and C. reticulata Diagnostic SNPs
Source: Front Plant Sci. 2019 Sep 24;10:1128. doi: 10.3389/fpls.2019.01128 (PMC6771394; doi:10.3389/fpls.2019.01128)
Supplement: Supplementary file 1 [file Table_1.docx]

Supplementary Material

Analysis of the phylogenic origin of key genes for citrus fruit quality using *C. maxima* and *C. reticulata* diagnostic SNPs

Milena do Amaral Santos^1^, Marcia Fabiana Barbosa de Paula^1^, Frederique Ollitrault^2^, Ronan Rivillan^3^, Edson Mario de Andrade Silva^1^, Abelmon da Silva Gesteira^4^, François Luro^5^, Dominique Garcia^3^, Patrick Ollitrault^2,*^, Fabienne Micheli^1,3,*^

*** Correspondence:** Patrick Ollitrault: [patrick.ollitrault@cirad.fr](mailto:patrick.ollitrault@cirad.fr); Fabienne Micheli: [fabienne.micheli@cirad.fr](mailto:fabienne.micheli@cirad.fr)

Supplementary Data

Supplementary Data 1. Examples of haplotype networks of carotenoid biosynthesis pathway established from resequencing data

For each gene, the two haplotypes of clementine were directly obtained from the reference sequence of an haploid Clementine (Phytozome) and the re-sequencing data of the diploid Nules clementine. Willow leaf mandarin and Ridge Pineaple sweet orange haplotypes were obtained from their re-sequencing data according to their parental relationship with clementine. Duncan grapefruit haplotypes were then deduced from its resequencing data tacking advantage of its parentalship with sweet orange. Haplotypes from Nadorcott, Ponkan and Cleopatra Mandarin as well as Chandler and acidless Pummelo and Sour orange were inferred from their re-sequencing data by statistical approach using GEVALT software (Ofir Davidovich, Gad Kimmel and Ron Shamir. GEVALT: An integrated software tool for genotype analysis. BMC Bioinformatics 2007, 8:36) integrated in SNiPlay (<http://sniplay.southgreen.fr/cgi-bin/home.cgi>). The haplotype networks were established with Haplophyle software integrated in SNiPlay.


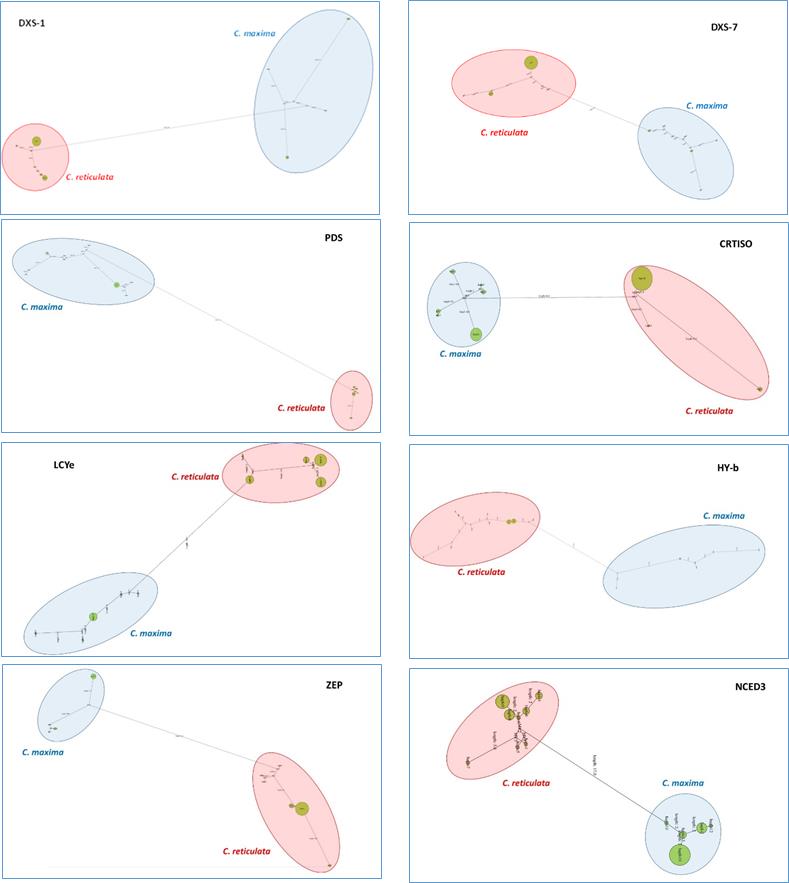


# Supplementary Tables

**Supplementary Table 1.** List of the 92 varieties used in this study. Variety name was according to Tanaka (1957). Pedigree from Ollitrault et al. (2012), Wu et al. (2014, 2018), Curk et al. (2016) and Oueslati et al., 2017).

Varieties used for the *in silico* analysis are indicated in bold. R: *C. reticulata*; M: *C. maxima*; C: citron *C. medica*; Mic.: *C. micrantha*; F1: hybrid.

| Horticultural group | Tanaka classification | Commun name | pedigree | Total of varieties |
| --- | --- | --- | --- | --- |
| Mandarins | *Citrus reticulata* | Citrus depressa mandarin | R/? | 22 |
|  |  | Nan feng mi chu mandarin | R/M |  |
|  |  | San Hu Hong Chu mandarin | R/M |  |
|  |  | Szibat mandarin | R/M |  |
|  |  | **Cleopatra mandarin** | R |  |
|  |  | Dancy mandarin | R/M |  |
|  |  | King mandarin | R/M |  |
|  |  | Ladu mandarin | R/M |  |
|  |  | **Ponkan mandarin** | R/M |  |
|  |  | Satsuma Owari mandarin | R/M |  |
|  |  | Sunki mandarin | R |  |
|  |  | **Willowleaf mandarin** | R/M |  |
|  |  | Kara mandarin | R/M |  |
|  |  | Carvalhal mandarin | R/M |  |
|  |  | Fairchild mandarin | R/M |  |
|  |  | Fortune mandarin | R/M |  |
|  |  | Fremont mandarin | R/M |  |
|  |  | Wilking mandarin | R/M |  |
|  |  | Fallglo mandarin | R/M |  |
|  |  | Se Hui Gan mandarin | R/M |  |
|  |  | Daoxianensis mandarin | R/M |  |
|  |  | Fuzhu mandarin | R/M |  |
| Pummelos | *Citrus maxima* | **Chandler pummelo** | M/R | 10 |
|  |  | Timor pummelo | M |  |
|  |  | Deep red pummelo | M |  |
|  |  | Kao Pan pummelo | M |  |
|  |  | Nam Roy pummelo | M |  |
|  |  | Pink pummelo | M |  |
|  |  | Tahiti pummelo | M |  |
|  |  | Azimboa pummelo | M |  |
|  |  | Da Xanh pummelo | M |  |
|  |  | Gil pummelo | M |  |
| Citrons | *Citrus medica* | Corsican citron | C | 5 |
|  |  | Etrog Citron | C |  |
|  |  | Mac Veu de montagne citron | C |  |
|  |  | Buddha’s hand citron | C |  |
|  |  | Hupang citron | C |  |
| Papeda | *C. micrantha* | Micrantha | Mic. | 1 |
| Clementina | *C. clementina* | Haploide Clementine | R/M | 2 |
|  |  | **Clementine Nules** | R/M |  |
| Sour orange | *C. aurantium* | **BigaradierMaroc** | F1: RxM | 2 |
|  |  | Bouquetier de Nice | F1: RxM |  |
| Sweet Orange | *Citrus sinensis* | Valencia late | R/M | 2 |
|  |  | **Washington navel** | R/M |  |
| Grapefruit | *Citrus paradisi* | **Duncan grapefruit** | R/M | 3 |
|  |  | Marsh grapefruit | R/M |  |
|  |  | Star Ruby grapefruit | R/M |  |
| Tangelo | *C. reticulata × C. paradisi* | Nova tangelo | R/M | 17 |
|  |  | Osceola tangelo | R/M |  |
|  |  | Page tangelo | R/M |  |
|  |  | Robinson tangelo | R/M |  |
|  |  | Allspice tangelo | R/M |  |
|  |  | Mapo tangelo | R/M |  |
|  |  | Minneola tangelo | R/M |  |
|  |  | Orlando tangelo | R/M |  |
|  |  | Pearl tangelo | R/M |  |
|  |  | Sampson tangelo | R/M |  |
|  |  | San Jacinto tangelo | R/M |  |
|  |  | Seminole tangelo | R/M |  |
|  |  | Sunrise tangelo | R/M |  |
|  |  | Sunshine tangelo | R/M |  |
|  |  | UGLI® tangelo | R/M |  |
|  |  | Webber tangelo | R/M |  |
|  |  | Wekiwa tangelo | R/M |  |
| Tangor | *C. reticulata × C. sinensis* | Temple tangor | R/M | 7 |
|  |  | Ambersweet tangor | R/M |  |
|  |  | Dweet tangor | R/M |  |
|  |  | Ellendale tangor | R/M |  |
|  |  | Kiyomi tangor | R/M |  |
|  |  | Murcott tangor | R/M |  |
|  |  | Ortanique tangor | R/M |  |
| Orangelo | *C. paradisi × C. sinensis* | Jackson orangelo | R/M | 2 |
|  |  | Triumph orangelo | R/M |  |
| *Lemons and limes* | *Citrus limon* | Lisbon lemon | (RxM)xC | 10 |
|  | *Citrus limon* | Eureka lemon | (RxM)xC |  |
|  | *Citrus meyeri* | Meyer lemon | (R/M)xC |  |
|  | *Citrus jambhiri* | Rough lemon | F1: RxC |  |
|  | *Citrus limonia* | Volkamer lemon | F1: RxC |  |
|  | *Citrus limonia* | Rangpur lime | F1: RxC |  |
|  | *Citrus limettioides* | Palestinian sweet lime | (RxM)xC |  |
|  | *Citrus limetta* | Marrakech limonette | (RxM)xC |  |
|  | *Citrus aurantiifolia* | Mexican lime | MicxC |  |
|  | *C. bergamia* | Bergamotte | R/M/C |  |
| *Nasnaran* | *C. amblycarpa* | Nasnaran | F1: MxMic. | 1 |
| Out-group | *Citrus hystrix* | Combava | - | 1 |
|  | *Poncirus trifoliata* | Pomeroy trifoliate | - | 2 |
|  |  | Rubidoux trifoliate | - |  |
|  | *Clymenia polyandra* | Clymenia polyandra | - | 1 |
|  | *Eremocitrus glauca* | Desert lime | - | 1 |
|  | *Microcitrus australasica* | Australian finger lime | - | 1 |
|  | *Citrus japonica* | Marumi kumquat | - | 1 |
|  | *Severinia buxifolia* | Chinese box orange | - | 1 |
| Total | | | | 92 |

**Supplementary Table 2.** Complete list of the genes used in this study. ID: identification in phytozome annotation of clementine reference genome (Wu et al., 2014).

| Metabolic pathway | Gene name | Gene code | ID |
| --- | --- | --- | --- |
| Carotenoid genes | Phytoene syntase | PSY_6 | Ciclev10011841m |
|  | Phytoene desaturase | PDS_9a | Ciclev10005632m |
|  |  | PDS_9b | Ciclev10007114m |
|  | Beta-carotene 3-hydroxylase | HYB_9 | Ciclev10005481m |
|  | Carotene cis-transisomerase | CRTISO_6 | Ciclev10011230m |
|  | Lycopene beta-cyclase | LCYb_9 | Ciclev10004730m |
|  | Capsanthin/capsorubin synthase | CCS_8 | Ciclev10028245m |
|  | Lycopene epsilon-cyclase | LCYe_1 | Ciclev10008410m |
|  | Zeaxanthin epoxidase | ZEP_7 | Ciclev10025089m |
|  | Zeta-carotene isomerase | Z-ISO_3 | Ciclev10020648m |
|  | Epoxycarotenoid dioxygenase | NCED_2 | Ciclev10014639m |
|  |  | NCED_9 | Ciclev10006710m |
|  |  | NCED_3 | Ciclev10019364m |
|  | 1-deoxy-D-xylulose-5-phosphate synthase | DXS_7 | Ciclev10024949m |
|  |  | DXS_9 | Ciclev10004432m |
|  |  | DXS_1 | Ciclev10007595m |
| Sugar genes | Fructan exohydrolase | FEH_1 | Ciclev10007827m |
|  | Invertase_beta-frutofuranosidase | INV_6 | Ciclev10013701m |
|  |  | INV_7a | Ciclev10025243m |
|  |  | INV_7b | Ciclev10025259m |
|  |  | INV_9 | Ciclev10004465m |
|  | Sucrose-phosphate phosphatase | SPP_2 | Ciclev10015425m |
|  |  | SPP_6 | Ciclev10011822m |
|  | SucrosePhosphateSynthase | SPS_1a | Ciclev10007312m |
|  |  | SPS_3 | Ciclev10018655m |
|  | Glycosyl transferase | GT_9 | Ciclev10004221m |
|  | Sucrose Synthase | SUSY_1a | Ciclev10010343m |
|  |  | SUSY_1b | Ciclev10007483m |
|  |  | SUSY_3 | Ciclev10018889m |
|  |  | SUSY_6 | Ciclev10011062m |
|  |  | SUSY_9 | Ciclev10004341m |
|  | Sucrose Transporter | SUT2_4 | Ciclev10030996m |
|  |  | SUT4_5 | Ciclev10000941m |
|  |  | SUT1_5 | Ciclev10000828m |
| Chlorophyll genes | Pheophorbide a oxygenase | PAO_8 | Ciclev10028147m |
|  | Geranylgeranyl diphosphate reductase | GDR_3 | Ciclev10020061m |
|  | Geranylgeranyl diphosphate reductase | GDR_2 | Ciclev10015206m |

Supplementary Table 3. Distribution of SNPs in PCA analysis. The same color indicated markers with the same segregation pattern.

| Marker code | Principal axis | | | Chrm. | |
| --- | --- | --- | --- | --- | --- |
|  | F1 | F2 | F3 | Number | Position |
| DXS_1_004 | -0.742 | 0.651 | -0.008 | 1 | 2201769 |
| DXS_1_005 | 0.898 | 0.314 | -0.030 | 1 | 2204655 |
| LCYe_1_016 | 0.756 | 0.334 | -0.117 | 1 | 10946683 |
| LCYe_1_018 | 0.247 | -0.015 | -0.243 | 1 | 10947735 |
| LCYe_1_017 | -0.756 | -0.334 | 0.117 | 1 | 10949337 |
| SPS_1b_118 | 0.925 | 0.326 | -0.003 | 1 | 20335430 |
| SUSY_1a_076 | 0.620 | 0.221 | -0.038 | 1 | 24769884 |
| SUSY_1a_074 | -0.925 | -0.321 | 0.033 | 1 | 24769925 |
| SUSY_1a_075 | -0.848 | -0.214 | -0.086 | 1 | 24770550 |
| SUSY_1b_077 | 0.764 | -0.125 | -0.124 | 1 | 24815532 |
| SUSY_1b_078 | -0.764 | 0.585 | 0.007 | 1 | 24815911 |
| SUSY_1b_079 | -0.810 | -0.299 | -0.102 | 1 | 24817862 |
| SPS_1b _065 | 0.924 | 0.349 | -0.005 | 1 | 25882871 |
| SPS_1b _117 | -0.673 | 0.658 | -0.135 | 1 | 25882940 |
| SPS_1b _119 | 0.742 | -0.651 | 0.008 | 1 | 25883715 |
| SPP_2_060 | 0.716 | -0.414 | 0.129 | 2 | 647558 |
| SPP_2_059 | 0.862 | 0.256 | -0.034 | 2 | 648813 |
| SPP_2_112 | 0.622 | -0.402 | 0.103 | 2 | 649874 |
| SPP_2_058 | 0.902 | 0.357 | 0.091 | 2 | 649938 |
| GDR_2_142 | -0.566 | 0.369 | -0.371 | 2 | 10232512 |
| GDR_2_141 | 0.473 | -0.138 | -0.037 | 2 | 10233106 |
| NCED_2_002 | -0.590 | 0.742 | 0.259 | 2 | 35236903 |
| NCED_2_100 | -0.553 | 0.707 | 0.134 | 2 | 35237027 |
| GDR_3_139 | -0.533 | 0.551 | -0.150 | 3 | 7269388 |
| GDR_3_137 | -0.710 | 0.174 | -0.314 | 3 | 7269418 |
| GDR_3_138 | -0.640 | 0.678 | -0.144 | 3 | 7269966 |
| SPS_3_070 | -0.815 | -0.441 | -0.066 | 3 | 23004267 |
| SPS_3_120 | -0.820 | -0.243 | -0.234 | 3 | 23007967 |
| SPS_3_121 | 0.874 | 0.238 | 0.106 | 3 | 23008019 |
| SPS_3_069 | -0.795 | 0.022 | 0.124 | 3 | 23008371 |
| NCED_3_028 | 0.714 | -0.672 | 0.037 | 3 | 29352238 |
| NCED_3_029 | -0.712 | 0.596 | -0.033 | 3 | 29353038 |
| NCED_3_030 | 0.549 | 0.204 | -0.370 | 3 | 29353389 |
| Z-ISO_3_041 | 0.897 | 0.336 | -0.051 | 3 | 39692943 |
| Z-ISO_3_105 | 0.722 | 0.077 | -0.165 | 3 | 39693203 |
| Z-ISO_3_042 | -0.907 | -0.344 | -0.022 | 3 | 39694029 |
| SUSY_3_080 | 0.728 | 0.239 | 0.270 | 3 | 46011973 |
| SUSY_3_125 | -0.754 | -0.168 | -0.423 | 3 | 46015516 |
| SUSY_3_082 | -0.718 | -0.308 | -0.233 | 3 | 46015618 |
| SUT2_4_090 | -0.854 | -0.162 | 0.131 | 4 | 23706202 |
| SUT2_4_089 | 0.765 | -0.205 | -0.174 | 4 | 23707455 |
| SUT2_4_091 | 0.607 | -0.676 | 0.077 | 4 | 23708831 |
| SUT2_4_129 | -0.632 | -0.086 | -0.277 | 4 | 23710311 |
| SUT4_5_093 | -0.655 | -0.280 | -0.589 | 5 | 35066730 |
| SUT4_5_092 | -0.704 | 0.499 | -0.050 | 5 | 35067373 |
| SUT4_5_094 | 0.655 | 0.280 | 0.589 | 5 | 35070565 |
| SUT1_5_096 | -0.917 | -0.337 | -0.021 | 5 | 39527580 |
| SUT1_5_095 | 0.894 | 0.281 | 0.024 | 5 | 39528405 |
| CRTISO_6_003 | 0.887 | 0.307 | -0.213 | 6 | 19580111 |
| CRITSO_6_001 | 0.653 | 0.227 | -0.495 | 6 | 19580386 |
| CRITSO_6_002 | 0.596 | -0.746 | -0.200 | 6 | 19581034 |
| SPP_6_063 | 0.249 | -0.524 | -0.293 | 6 | 19587634 |
| SPP_6_061 | -0.887 | -0.307 | 0.213 | 6 | 19587935 |
| SPP_6_115 | -0.637 | -0.224 | 0.396 | 6 | 19588948 |
| SPP_6_116 | -0.853 | -0.248 | 0.074 | 6 | 19589174 |
| INV_6_048 | 0.560 | 0.010 | -0.448 | 6 | 20334510 |
| INV_6_107 | -0.211 | -0.580 | 0.014 | 6 | 20334875 |
| INV_6_108 | -0.861 | -0.287 | 0.083 | 6 | 20335430 |
| INV_6_047 | 0.886 | 0.306 | -0.017 | 6 | 20336709 |
| PSY_6_143 | 0.793 | 0.106 | -0.365 | 6 | 21391650 |
| PSY_6_144 | 0.793 | 0.106 | -0.365 | 6 | 21393272 |
| PSY_6_145 | 0.523 | -0.354 | 0.018 | 6 | 21393302 |
| SUSY_6_085 | 0.006 | 0.458 | 0.416 | 6 | 21405852 |
| SUSY_6_083 | -0.881 | -0.316 | 0.219 | 6 | 21406297 |
| SUSY_6_084 | -0.596 | 0.746 | 0.200 | 6 | 21407171 |
| ZEP_7_104 | -0.401 | 0.396 | -0.196 | 7 | 3222750 |
| ZEP_7_38 | -0.635 | 0.655 | -0.033 | 7 | 3223699 |
| ZEP_7_102 | 0.656 | -0.710 | -0.024 | 7 | 3223842 |
| ZEP_7_039 | -0.707 | 0.619 | -0.010 | 7 | 3228529 |
| DXS_7_097 | 0.710 | 0.083 | -0.085 | 7 | 5043747 |
| DXS_7_007 | -0.791 | 0.065 | 0.119 | 7 | 5044084 |
| DXS_7_008 | -0.683 | 0.684 | -0.082 | 7 | 5044407 |
| INV_7a _050 | -0.917 | -0.324 | -0.009 | 7 | 6557846 |
| INV_7a _049 | 0.848 | 0.148 | -0.107 | 7 | 6558776 |
| INV_7a _051 | -0.620 | -0.263 | 0.407 | 7 | 6559831 |
| INV_7b_052 | 0.757 | -0.169 | 0.187 | 7 | 6562070 |
| INV_7b _054 | 0.526 | 0.228 | -0.381 | 7 | 6562309 |
| INV_7b _053 | 0.706 | -0.593 | 0.013 | 7 | 6562761 |
| CCS_8_019 | -0.678 | 0.349 | -0.091 | 8 | 19259940 |
| CCS_8_021 | 0.665 | -0.488 | 0.194 | 8 | 19260511 |
| CCS_8_020 | 0.712 | 0.202 | 0.015 | 8 | 19260867 |
| PAO_8_133 | 0.835 | 0.183 | -0.119 | 8 | 21113191 |
| PAO_8_134 | -0.842 | -0.223 | 0.117 | 8 | 21116731 |
| PAO_8_135 | -0.019 | -0.482 | -0.194 | 8 | 21116878 |
| SUSY_9_086 | 0.817 | 0.264 | -0.156 | 9 | 1499940 |
| SUSY_9_087 | 0.923 | 0.336 | -0.018 | 9 | 1502120 |
| SUSY_9_088 | 0.795 | 0.290 | -0.216 | 9 | 1502352 |
| DXS_9_011 | -0.913 | -0.355 | 0.004 | 9 | 2354611 |
| INV_9_110 | -0.280 | 0.308 | -0.036 | 9 | 2355144 |
| DXS_9_010 | 0.890 | 0.347 | 0.075 | 9 | 2355144 |
| DXS_9_012 | 0.742 | -0.651 | 0.008 | 9 | 2355446 |
| GT_9_073 | -0.916 | -0.327 | 0.005 | 9 | 15985569 |
| GT_9_124 | 0.622 | 0.149 | -0.047 | 9 | 15987011 |
| GT_9_123 | 0.912 | 0.279 | -0.080 | 9 | 15987291 |
| PDS_9b _035 | -0.916 | -0.327 | 0.005 | 9 | 17778673 |
| PDS_9b _036 | -0.830 | -0.116 | 0.094 | 9 | 17779170 |
| PDS_9b _037 | -0.246 | -0.156 | 0.000 | 9 | 17779554 |
| PDS_9a _032 | -0.916 | -0.327 | 0.005 | 9 | 17790595 |
| PDS_9a _033 | 0.916 | 0.327 | -0.005 | 9 | 17791304 |
| PDS_9a _034 | 0.626 | -0.561 | -0.007 | 9 | 17792958 |
| LCYb_9_022 | -0.916 | -0.327 | 0.005 | 9 | 22728581 |
| LCYb_9_023 | 0.916 | 0.327 | -0.005 | 9 | 22728720 |
| LCYb_9_024 | -0.576 | 0.517 | 0.005 | 9 | 22729397 |
| INV_9_111 | -0.279 | 0.303 | -0.029 | 9 | 27283782 |
| INV_9_055 | 0.621 | -0.306 | 0.338 | 9 | 27286067 |
| INV_9_056 | -0.811 | -0.105 | -0.021 | 9 | 27287227 |
| HYB_9_099 | 0.742 | -0.651 | 0.008 | 9 | 29488796 |
| HYB_9_013 | -0.742 | 0.651 | -0.008 | 9 | 29488937 |
| HYB_9_014 | 0.353 | -0.315 | -0.012 | 9 | 29489930 |
| HYB_9_015 | -0.689 | -0.285 | -0.539 | 9 | 29490755 |

Supplementary Table 4. Diagnostic set of 115 SNPs related to fruit quality and their impact on the protein sequence. aa: amino acid; R: *C. reticulata*; M: *C. maxima*. See Excel File.

Supplementary Table 5. Phylogenetic origin of 29 genes of the carotenoid, sugar and chlorophyll biosynthesis pathways for 75 citrus varieties. See Excel File.
